# Completion thyroidectomy: predicting bilateral disease

**DOI:** 10.1186/s40463-015-0076-4

**Published:** 2015-06-16

**Authors:** Badr Ibrahim, Véronique-Isabelle Forest, Michael Hier, Alex M. Mlynarek, Derin Caglar, Richard J. Payne

**Affiliations:** Division of Otolaryngology Head and Neck Surgery, McGill Thyroid Cancer Center, Jewish General Hospital, 3755 Cote Ste Catherine, Montreal, H3T 1E2 QC Canada; Jewish General Hospital – Otolaryngology Head and Neck Surgery Clinic, 3755 Côte-Sainte-Catherine Road, Montreal, H3T 1E2 QC Canada; Department of Pathology, McGill University Health Center Glen site, 1001 Boulevard Decarie, Montreal, H4A 3J1 QC Canada

**Keywords:** Completion thyroidectomy, Thyroid carcinomas, Bilateral disease, Decision making, Patient information

## Abstract

**Introduction:**

It is not uncommon for patients with indeterminate thyroid nodules to undergo diagnostic hemithyroidectomy. When the final pathology determines that the nodule is in fact malignant, patients require counseling as to the whether a completion thyroidectomy is necessary.

**Objectives:**

1. Determine the incidence of well differentiated thyroid cancer (WDTC) in the contralateral thyroid lobe in patients undergoing completion thyroidectomy.
2. Identify features of the malignant tumor in the initial resection that increase the likelihood of malignancy in the contralateral lobe.

**Methods:**

Retrospective chart review of 97 patients who underwent hemithyroidectomy and completion thyroidectomy in a university’s teaching hospital network between 2006 and 2012. Pathology reports from both surgeries as well as patient and thyroid nodule characteristics were reviewed.

**Results:**

Of the 97 patients, 47 (48 %) had a malignancy in the contralateral lobe. In the contralateral lobe, 42/47 (89 %) of malignancies were papillary microcarcinomas (PMC) and 15/42 (36 %) of the PMC were multifocal. Multifocal malignancies in the initial specimen had a 60 % rate of contralateral malignancy and were found to be a predictor of bilateral disease (*p* = 0.04) with *OR* = 2.74 (95 % CI: 1.11–6.79; *p* = 0.003) in WDTC and *OR* = 3.59 (95 % CI:1.35 9.48; *p* = 0.01) in papillary cancer specifically. There was no statistical significant correlation established for the following variables: presence of positive cervical nodes, extrathyroidal extension, positive resection margins, size and angio-lymphatic invasion. Moreover, there was no statistical correlation between any of the variants of papillary thyroid cancer and bilateral disease, even though most aggressive subtypes were found to be bilateral.

**Conclusion:**

In this study, the rate of malignancy in the contralateral lobe was 48 %. Multifocality and presence of an aggressive subtype of papillary thyroid cancer in the initial specimen were found to be more important variables to consider in decision-making regarding completion thyroidectomy than size of the initial tumor alone.

## Background

Thyroid nodules are commonly encountered in clinical practice. Palpable nodules have a prevalence ranging from 5.3 to 6.4 % in women and 0.8 to 1.5 % in men in iodine sufficient regions and an incidence rate of 1.7 % in women and 0.9 % in men. Their prevalence increases with age up to 9.1 % in women older than 75 years old (1, 2). While the majority of these nodules are benign, a thyroid carcinoma will be diagnosed in about 5 % of cases [1, 2]. In their 2009 revised guidelines for the management of patients with thyroid nodules and differentiated thyroid cancer, the American Thyroid Association recommends ultrasonography followed by fine needle aspiration biopsy (FNAB) as diagnostic modalities in the work up of thyroid nodules [3]. However, 15–30 % of FNAB will be indeterminate (3) leaving the physician and the patient unable to determine with certainty the risk of malignancy and therefore, the need for surgery. As a matter of fact, it has been reported in the literature that the risk of malignancy of a nodule with an indeterminate FNAB ranges from 12 to 42 % [4–8]. For that reason, the 2009 ATA guidelines recommend a diagnostic thyroid lobectomy for nodules with indeterminate FNAB as an initial surgical approach [3]. If the definitive diagnosis confirms that the lesion is malignant, they suggest that patients undergo a completion thyroidectomy unless the lesion was <1 cm in size, unifocal, intrathyroidal, without lymph node involvement, and a low risk tumor [3].

The main reasons for completion thyroidectomy are to optimize radioiodine ablation of the remaining thyroid tissue, to allow for better postoperative follow up of thyroglobulin levels with the aim of detecting recurrences, as well as to obtain a complete excision of multifocal disease. Currently, there are no preoperative, intraoperative or postoperative definitive features or methods to ascertain the presence of disease in the contralateral lobe. Therefore, the pathological analysis of the hemithyroidectomy becomes the cornerstone of the decision making process in advising the patients on the need for completion thyroidectomy. This study aims at identifying the rate of bilateral disease on completion thyroidectomy and finding predictors for the presence of malignancy in the contralateral lobe.

## Methods

### Patient selection

After obtaining the combined approval from the McGill University Health Center and Jewish General Hospital Ethics boards for conducting this retrospective chart review, we proceeded with pooling the operating room records of one surgeon from August 2006 to April 2012 of cases of thyroid surgeries. From the 1264 thyroid surgery cases, 115 patients had a completion thyroidectomy. However, after exclusion of patients with incomplete electronic charts and one case where the initial pathology was benign, 97 cases were included in the present study. These patients had undergone a hemithyroidectomy followed by a completion thyroidectomy for malignancy identified in the first resected lobe.

### Data collection

Baseline demographic information and dates of surgeries were recorded to calculate age at time of surgery and intervals between surgeries. The McGill Thyroid Nodule Score (MTNS) was recorded, when available, for each patient’s initial nodule. The MTNS is a comprehensive assessment tool previously developed by our research group consisting of a 22 variable scoring system that takes into account clinical parameters and laboratory values, imaging results, and cytology. It has been shown to be an accurate predictor of the risk of malignancy in a given thyroid nodule [9].

Pathology reports of both surgeries were reviewed. Patients were divided into two groups (benign contralateral lobe vs malignant contralateral lobe) based on the results of the final pathology report of the completion thyroidectomy specimen. The histological type of the tumor and when relevant, the variant of papillary thyroid carcinoma (PTC), were collected. Also, size, tumor capsule status, lymphovascular involvement, extrathyroidal extension, lymph node metastasis, resection margins, multifocality and number of PTC and papillary microcarcinomas (PMC) were recorded. A PMC was defined as a focus of papillary carcinoma less than 1 cm in size. In this study, the presence of an isolated focus of PMC was recorded as a malignant specimen, and multifocality was defined as the presence of more than one malignant focus in the resected thyroid lobe, regardless of size. The presence of a lymph node metastasis was not counted as multifocal disease but rather as a lymph node metastasis. Extrathyroidal extension refers to a tumor that extends into the perithyroidal tissue or surrounding soft tissue (excluding lymph node metastasis). A patient with a malignant tumor in each lobe was accounted for as a case of bilateral disease without necessarily having the same histologic type of tumor in each lobe. Details pertaining to other histopathological changes such as chronic lymphocytic thyroiditis were not recorded and a specimen with no cancerous focus identified was recorded as benign.

### Statistical analysis

Categorical variables were assessed by means of a Chi-Square test. Age, time to completion, and the MTNS were tested with the student *T* test. Statistical outliers in time to completion were identified by means of a Modified Z-score. A two-tailed Fisher’s exact test was used to assess the relation between different papillary carcinoma variants and bilateral disease. Binary logistic regressions were used to confirm the independent correlations with bilateral disease in subsets of our sample. The calculations were made using SPSS statistics version 21.

## Results

### Patients demographics

From the 97 patients identified, 77 (79.4 %) were females equally distributed between the group with a benign contralateral lobe and the group with bilateral disease. In comparison only 20 (20.6 %) patients were males with 11 having a benign contralateral lobe and 9 having malignancy. The average age of both groups at the time of the initial surgery was similar: 47.3(±12.9) for the group with a benign lobe on completion and 46.7(±15.1) for the group with a malignant contralateral lobe. The average interval between the initial surgery and the completion thyroidectomy varied for both groups with 73.2(±21.5) days in the benign completion group and 84.2(±24.2) in the malignancy group (*p* = 0.05) (Table 1).

**Table 1:** Patient demographics

Total *n* = 97	Absence of malignancy in the contralateral lobe (*n* = 50)	Presence of malignancy in the contralateral lobe (*n* = 47)	p values
Gender			
Males	11	9	0.73
Females	39	38	
Mean Age at Initial Resection (±SD)	47.6(±12.9)	46.3(±15.0)	0.64
Mean Time To Completion in Days (±SD)	73.2(±21.5)	84.2(±24.2)	0.05

Sixteen of the excluded patients had an incomplete chart missing whole or part of the pathology report from the first surgery. All of them however had a completion surgery pathology report. One patient had a total thyroidectomy misclassified as a completion surgery in the database. The average age of the excluded patients was 55.6 years. They were composed of 16 females and 1 male and had a 65 % malignancy rate at completion (*n* = 11/17). Thirty five percent (*n* = 6/17) of these tumors were multifocal.

### Pathology

In the specimen of the hemithyroidectomy of the initial resection, 75 (77.3 %) nodules exhibited PTC, 14 (14.4 %) PMC (of which 9 (64 %) where multifocal), 6 (6.3 %) follicular carcinoma, 1 (1.0 %) medullary microcarcinoma, and 1 (1.0 %) Hurthle cell carcinoma. The distribution of the remaining pathological features recorded after the initial resection is shown in Table 2.

**Table 2:** Features of patients with malignant specimens at first surgery and correlation to bilateral disease at completion

*N* = 97		Unilateral (*n* = 50)	Bilateral (*n* = 47)	p
Age ≥45	Yes	29	23	0.42
	No	21	24	
Female Gender	Yes	39	38	0.81
	No	11	9	
Lymphovascular Involvement	Yes	6	7	0.77
	No	44	40	
Ipsilateral Lymph Nodes	Yes	9	11	0.62
	No	41	36	
Extrathyroidal Extension*	Yes	8	9	0.79
	No	41	37	
High Risk Variants of PC	Yes	1	4	0.36
	No	36	35	
Positive Resection Margin**	Yes	13	9	0.47
	No	36	38	
Tumor Capsule Involvement	Yes	6	5	1
	No	18	17	
	NA	25	26	
Multifocality	Yes	19	28	0.04
	No	31	19	
Histology	PC	38	37	0.13
	PMC	5	9	
	FOLCA	5	1	
	Other	2	0	
Size(cm)	0–0.9	6	9	0.72
	1–1.9	12	10	
	2–2.9	14	10	
	>3	18	18	

*n = 95 because 2 could not be assessed on pathologic examination; **n = 96 because 1 could not be assessed on pathologic examination

p calculated by Chi-square test

In the completion thyroidectomy specimen, from the 97 cases 46 had a malignancy in the contralateral lobe and 1 case had a malignancy only in a lymph node on the contralateral side for a total of 47 (48 %) malignant cases. Forty-two (43 %) had a PMC, of which 15 (36 %) were multifocal, and 5 (5 %) had a PTC (4 intra-thyroidal and 1 involving a lymph node only) none of which were multifocal. The remaining 50 (52 %) cases exhibited a benign contralateral lobe. As far as lymph node status is concerned, out of the 47 cases of intrathyroidal tumor involvement, 7 (15 %) exhibited malignant contralateral lymph nodes. These 7 cases were constituted of 2 cases with multifocal PMC, 1 unifocal PMC, and 4 cases of PTC in the thyroid gland.

As for extrathyroidal extension (ETE) in the completion specimens, there were 2 cases of PTC with ETE while there was one case of unifocal PMC with ETE.

### Predictors of bilateral disease

Our initial screening of the different variables showed that only multifocality had a statistically significant correlation with bilateral disease (Table 2). To assess the independence of this correlation and to control for the possible impact of the other studied features on the presence of bilateral disease in well-differentiated thyroid cancers, a binary logistic regression was done using the following variables: age over 45, female gender, histology, size of the tumor, lymphovascular invasion, lymph node involvement, extrathyroidal extension, tumor capsule involvement and multifocality. Multifocality in the initial specimen was the only variable independently associated with bilateral disease with an OR = 2.74 (95 % CI =1.11–6.79; *p* = 0.03) (Table 3). When analyzing only PTC and PMC cases, multifocality in the initial specimen becomes an even stronger predictor with an OR = 3.59 (95 % CI = 1.36–9.48; *p* = 0.01) (Table 4). Furthermore, in this subgroup, the involvement of ipsilateral lymph nodes at the initial surgery was not a predictor of bilateral disease [OR = 0.80 (95 % CI = 0.24–2.74; *p* = 0.73)] (Table 4). Also, as shown in Table 5, four out of the 5 cases of high risk variants of PTC (1 sclerosing, 3 tall cells and 1 solid variant) were associated with bilateral disease with OR = 4.00 (95 % CI = 0.43–37.6; *p* = 0.22). However, this correlation was not statistically significant. None of the low-risk variants were statistically significantly associated with bilateral disease (Table 5).

**Table 3:** Odds ratios of candidate predictors of bilateral disease following binary logistic regression in well differentiated cancers

*N* = 93^a^	Odds ratio	95 % confidence interval	p Value
Age ≥ 45	0.59	0.24–1.46	0.26
Female gender	0.76	0.25–2.30	0.63
Histology	0.63	0.26–1.54	0.31
Size	0.78	0.48–1.25	0.30
Lympho vascular invasion	1.49	0.33–6.65	0.60
Lymph node involvement	1	0.30–3.26	0.99
Extra thyroidal extension	1.06	0.30–3.73	0.93
Encapsulated tumor	0.90	0.52–1.56	0.69
Multifocality	2.74	1.11–6.79	0.03

R square = 0.111

^a^2 patients could not be included in this analysis as extra thyroidal extension could not be assessed on the pathology specimen

**Table 4:** Binary logistsic regression of candidate predictors of bilateral disease in papillary and micropapillary cancer subset

*N* = 87^a^	Odds ratio	95 % confidence interval	p Value
Age ≥ 45	0.48	0.18–1.28	0.14
Female Gender	0.62	0.19–2.05	0.43
Histology	1.95	0.29–13.3	0.49
Size	0.91	0.50–1.65	0.75
Lympho vascular invasion	1.78	0.37–8.64	0.46
Lymph node involvement	0.80	0.23–2.75	0.72
Extra thyroidal extension	1.08	0.29–3.94	0.91
Tumor capsular involvement	1.01	0.56–1.84	0.97
Multifocality	3.59	1.36–9.48	0.01

R square = 0.155

^a^2 patients could not be included in this analysis as extra thyroidal extension could not be assessed on the pathology specimen

**Table 5:** Papillary cancer variants and bilateral disease

Total *n* = 76	Absence of malignancy in the contralateral lobe (*n* = 37)	Presence of malignancy in the contralateral lobe (*n* = 39)
Oncocytic	6	5
Classical	2	6
Follicular	24	23
Macrofollicular	3	1
Hyalinizing Trabecular	1	0
Solid	1	0
Sclerosing	0	1
Tall Cell	0	3

## Discussion

In our present series, the rate of malignancy in the remaining lobe of the thyroid of patients undergoing completion thyroidectomy following a hemithyroidectomy positive for cancer is 48 % with close to 90 % of those tumors in the contralateral lobe being PMC. Among the variables analyzed, multifocality of the tumor in the initially resected lobe is the only feature that is consistently associated with bilateral disease [OR 2.74(95 % CI = 1.11–6.79; *p* = 0.03) in well differentiated cancers and 3.59 (95 % CI = 1.36–9.48; *p* = 0.01) in papillary cancers specifically]. In the literature, the incidence of bilateral carcinoma following CT ranges from 29 to 56.3 % [10–16] placing our rate of 48 % within this range (Table 6). More specifically, when considering only the patients with multifocal disease in the first resected lobe, the rate of bilateral malignancies is increased to 60 % as compared to 48 % in our entire series.

**Table 6:** Literature review of features predicting contralateral disease in well differentiated thyroid cancers

	Pasieka et al. 1992 [10] *N* = 60	Kawaura et al. 2001 [11] *N* = 128	Pacini et al. 2001 [12] *N* = 182	Alzahrani et al. 2002 [13] *N* = 101	Kim et al. 2004 [14] *N* = 81	Grigsby et al. 2006 [15] *N* = 150	Pitt et al. 2009 [16] *N* = 228^b^
Malignancy on completion	53 %	56.3 %	44 %	51.5 %	36 %	41 %	29 %
Multifocality	88 % (*n* = 14/16)	76.6 % (*n* = 36/47)	NC	53.5 % (*n* = 15/28)	69 % (*n* = 20/29)	----	45 % (*n* = 9/20)
	*p* < 0.001	*p* > 0.05		*p* = 0.045	*p* < 0.001		*p* = 0.02
Size of Tumor	NC	NC	NC	NC	NC	NC	NC
Lymph node involvement	NC	76.5 % (*n* = 13/17)	73.3.% (*n* = 11/15)	----	NC	NC	NC
		*p* > 0.05	*p* = 0.03				
Age	----	NC	NC	^a^60.9 % (*n* = 14/23)	NC	NC	----
				*p* = 0.01			
Gender	----	NC	NC	NC	----	NC	----
Thyroiditis	----	NC	----	----	----	----	----
Exposure to ionizing radiation	----	100 % (*n* = 6/6)	----	----	----	----	----
		*p* > 0.05					
Low vs High Risk	----	----	NC	----	----	NC	----
Histopathologic Diagnosis	NC	----	NC	----	NC	----	----
Coexistent Benign Nodule	----	----	----	----	NC	----	NC
Extrathyroidal Extension	----	NC	----	^a^61.5 % (*n* = 16/26)	NC	----	NC
				*p* = 0.01			
Serum Tg > 20 ng/ml	----	----	----	56.7 % (*n* = 17/30)	----	----	----
				*p* = 0.026			
PC variant	----	NC (Tall cell)	----	----	----	NC (Follicular)	NC (Follicular)
Soft tissue invasion	----	----	----		----	NC	----
Vascular invasion	----	----	----		----	NC	NC
Resection margins	----	----	----		----	NC	NC
Tumor capsular invasion	----	----	----	----	----	----	NC

---- Feature not studied, *NC* No statistically significant correlation

^a^Predictive of cervical lymph node metastasis only

^b^Pitt et al. [16]: the study of factors predicting contralateral disease is limited to PMC (<1 cm) *n* = 70

Furthermore, our sample demonstrates that size did not correlate with bilateral disease. Considering our previous finding regarding the independent correlation between bilateral disease and multifocality and knowing that 64 % of PMC found on initial resection were multifocal, it is probably clinically more important to consider the focality of the tumor rather than its size in the decision of proceeding with completion thyroidectomy. Indeed, the results of our study are in line with the ATA guidelines on the management of well differentiated thyroid cancers according to which completion thyroidectomy should be offered to all patients with thyroid cancer unless the tumor is 1) < 1 cm in size *2) unifocal* 3) intrathyroidal 4) node negative 5) low risk tumor [3], and underlines the need for all 5 conditions to be met simultaneously in order to decide not to proceed with completion thyroidectomy. Moreover, 43 % of the lesions that were found on completion in our data were PMC and 36 % of them were multifocal. Mantinan et al. followed 91 patients with incidentally found PMC during thyroid surgery for an average of 10 years in an effort to identify predictors of outcomes of incidental PMC. The only independent predictor of recurrence identified in this series was multifocality [17]. This further underscores the importance of multifocality in deciding on the extent of surgery.

As well, we did not perform intra-operative frozen sections to help guide our decision-making regarding the extent of surgery as this practice has been tried several years ago at our institution and did not prove useful. This is in line with multiple series in the literature that prove that frozen sections were of little clinical usefulness [18–20].

Regarding the involvement of cervical lymph nodes at the initial surgery, 52.3 % of patients with involved lymph nodes had disease in the contralateral lobe. Even if more than half of the patients with positive lymph nodes had bilateral disease, lymph node status was not a statistically significant predictor of bilateral disease in our series. On the other hand, Pacini et al. showed that, in a cohort of 182 patients, 73.3 % of patients with cervical lymph node involvement at the initial surgery had bilateral malignancy [12]. However, it is well established that in the presence of involved cervical lymph nodes a total thyroidectomy is warranted to help with radioactive iodine treatment and further follow-up [3]. For this reason, at our center, to determine the status of lymph node involvement and also, in an attempt to avoid subsequent surgery, we routinely perform sentinel lymph node biopsy using the methylene blue technique [21].

High risk variants have been shown to be more aggressive than classical PTC [22] and it is believed that anaplastic carcinoma develops from previously well differentiated thyroid cancer with tall cell variant being the most common coexistent carcinoma [23]. In fact, a recent review of more than 43,000 cases of papillary thyroid cancer aimed at identifying the prognostic factors for survival in tall cell and diffuse sclerosing subtypes of PTC showed that the incidence of these high risk variants is on the rise and that they are associated with significantly higher disease specific mortality rates [22]. Interestingly, it also showed that most diffuse sclerosing cases are < 1 cm in size and that their survival was unaffected by size. In our study, 4 out of 5 cases with high-risk variants had malignancy in the contralateral lobe. This finding did not reach statistical significance, but we believe that it is due to the small sample size. With thyroid surgery and RAI therapy being independently associated to improved outcome, the authors recommend aggressive treatment with both modalities in all cases of tall cell and diffuse sclerosing subtypes –regardless of size. Indeed, it is of common practice in our center to complete the thyroidectomy in patients with high-risk variants of papillary carcinoma.

This study did not demonstrate that the MTNS could be used as a predictor of bilateral disease. This is not a surprising finding as the MTNS was designed for and is effective to preoperatively predict the likelihood of malignancy in a given thyroid nodule; It was not meant to predict the risk of bilateral disease.

This study represents the second Canadian retrospective review of predictors of malignancy in the second lobe of the thyroid gland at completion surgery. Compared to the data presented by Kawaura et al. [11] in the early 2000’s, our series reaches statistical significance for the relationship between multifocal disease and bilateral malignancies. This, along with the other clinically significant associations outlined in this paper, can be used to improve communication between surgeons and patients regarding the risk of malignancy in the contralateral lobe of the thyroid and helps guiding both parties in the decision making process for the need and extent of future surgeries.

Lastly, our retrospective study may have been subject to selection bias. However, we had a good sample size and we have subjected our data to multivariate logistic regression to decrease the effect of bias.

## Conclusion

The rate of malignancy in the contralateral lobe of the thyroid gland following completion thyroidectomy is 48 %. When multifocal disease is found in the first resected lobe, the incidence of malignancy in the contralateral lobe increases to 60 %. Our data shows that multifocality and presence of an aggressive subtype of papillary thyroid cancer in the initial specimen were found to be more important variables to consider in decision-making regarding completion thyroidectomy than size of the initial tumor alone.
